# Epidemics, Public Sentiment, and Infectious Disease Equity Market Volatility

**DOI:** 10.3389/fpubh.2021.686870

**Published:** 2021-05-14

**Authors:** Jinxia Meng, Qingyi Su, Jinhua Zhang, Li Wang, Ruihui Xu, Cheng Yan

**Affiliations:** ^1^Jiaxing Vocational and Technical College, Jiaxing, China; ^2^Institute of World Economics and Politics, Chinese Academy of Social Sciences, Beijing, China; ^3^School of Economics, Zhejiang University of Technology, Hangzhou, China; ^4^Research Institute of the People's Bank of China (PBC), Beijing, China; ^5^Essex Business School, University of Essex, Colchester, United Kingdom

**Keywords:** COVID-19, public sentiment, infectious disease equities, epidemic, confirmed cases

## Abstract

**Background:** This article studies the relationship between the COVID-19 epidemic, public sentiment, and the volatility of infectious disease equities from the perspective of the United States. We use weekly data from January 3, 2020 to March 7, 2021. This provides a sufficient dataset for empirical analysis. Granger causality test results prove the two-way relationship between the fluctuation of infectious disease equities and confirmed cases. In addition, confirmed cases will cause the public to search for COVID-19 tests, and COVID-19 tests will also cause fluctuations in infectious disease equities, but there is no reverse correlation. The results of this research are useful to investors and policy makers. Investors can use the number of confirmed cases to predict the volatility of infectious disease equities. Similarly, policy makers can use the intervention of retrieved information to stabilize public sentiment and equity market fluctuations, and integrate a variety of information to make more scientific judgments on the trends of the epidemic.

## Introduction

Since the initial outbreak of COVID-19, this global epidemic has spread rapidly. According to data from Johns Hopkins Coronavirus Resource Center on March 23, 2021, the global number of Coronavirus cases has reached 123.6 million, of which the United States accounts for 29.9 million. Because the virus is highly contagious, countries have adopted strict quarantines, resulting in the forced closure of a large number of commercial activities. According to the U.S. Bureau of Labor Statistics, the unemployment rate in the United States in April 2020 was as high as 14.7%. Previous outbreaks of infectious diseases such as SARS and MERS-CoV did not have such a strong impact on the equity market as the COVID-19 pandemic. This shows that in response to the current coronavirus epidemic, government restrictions on business activities and stay-at-home policies have had a direct negative impact on the service-oriented economy. This is the main reason why the U.S. equity market's response to COVID-19 is stronger than its response to previous pandemics.

Many empirical studies have shown that various direct and indirect factors, such as the epidemic situation and investor attention, play an important role in equity market volatility. Compared with other sectors in the economy and financial system, the equity market will respond more directly to epidemics such as COVID-19. Li et al. (1) and Mazur et al. (2) have examined the impact of COVID-19 on U.S. and European equity markets. However, most of these authors examined the impact of the number of COVID-19 cases and deaths on the equity market and seldom examined the relationship between public sentiment, the epidemic, and equity market volatility.

Information epidemiology has become a research hotspot in the context of the spread of the COVID-19 epidemic (3). This area of research involves scanning the Internet, traditional media, and other public channels to obtain health-related data and content. In recent years, scholars have used the data collected by Google Trends and Google Flu Trends to conduct much of their research. Google Trends shows the keywords that the public searches using Google. The data is normalized according to search frequency and displayed in relative search volume. Data can be selected in different regions and time periods according to needs. Researchers can use Google Trends data to investigate people's search needs for coronavirus information around the world, and can choose five keywords for comparative analysis each time. This data is especially useful for studying seasonal infectious diseases, mental health conditions, and other diseases. This article uses Google Trends to analyze the public's judgment and information needs on epidemic trends.

It is generally believed that industries related to people's livelihoods, such as healthcare, food, software and technology, and natural gas, are performing better during the epidemic. The negative impact of the epidemic on the real estate, aviation, hotel, tourism, and entertainment industries is even more pronounced. So, what is the impact of COVID-19 on the volatility of infectious disease equities? To examine equity market volatility, Baker et al. (4) constructed a newspaper-based infectious disease equity market volatility tracker, which is different from traditional equity market volatility indicators. The data spans January 1985 to the present, and the data frequency is updated once a day. In contrast, our article is not only based on Google Trends, but also uses the volatility of infectious disease equities constructed based on traditional newspaper media to examine the relationship between the epidemic, public sentiment, and the volatility of infectious disease equities. This is of great significance to investors and decision makers.

## Literature Review

Research on COVID-19 has been extensive, and most studies consider disease-related keywords that the public searched for on Google during the epidemic, such as skin diseases, quitting smoking, and washing hands. Kutlu (5) uses Google Trends to judge the trends of skin diseases in Turkey and Italy during the COVID-19 pandemic. The study found that from March 11 to June 1, 2020, there was a statistically significant positive correlation between the number of COVID-19 cases and the search terms of general dermatology in Turkey. The search terms for “hair loss” and “acne” in these two countries increased during the COVID-19 epidemic. This may be related to emotional stress, anxiety, and depression. The increasing number of “acne” search terms in Google Trends may be related to the curfew and other blockade measures imposed on young people in Turkey and Italy. In addition, the widespread use of masks may also cause acne. Interestingly, during the COVID-19 pandemic, the significant reduction in sexually transmitted disease search terms may be related to the fact that social distancing, gatherings, and stay-at-home campaigns have led to a decrease in extramarital sexual activity. In addition, in the summer, the closure of many tourist centers led to a decrease in the search term “sunscreen.” The article points out that understanding the trends of skin diseases and the impact on public perception during the COVID-19 pandemic will help dermatologists better prepare.

Springer et al. (6) believes that people's searches on the Internet are mainly based on rational information needs and demographic needs in order to prepare for the pandemic and to protect themselves. This includes terms such as “hand washing” and “social distancing.” This reflects an increase in people's fear of infection. These search terms all reflect global attention. According to Strzelecki (7), the peak time for new cases occurs within 10–14 days after the keyword peaks of search terms such as “COVID-19 symptoms,” “social distance,” and “isolation.” Heerfordt and Heerfordt (8) points out that smokers are not only more susceptible to flu and Middle East respiratory syndrome and other coronavirus infectious diseases, but the consequences are also more serious. Studies have found that among hospitalized patients with COVID-19, smokers are two to nine times more likely to have serious complications than non-smokers. Quitting smoking can not only reduce respiratory symptoms and bronchial responsiveness, but also effectively prevent lung function decline. Walker et al. (9) show that there is a significant correlation between the use of search terms related to “odor” and the number of coronavirus cases and deaths. This correlation exists widely in the sample countries examined. Generally, the detection of the first coronavirus death is significantly consistent with the time of the outbreak in the country. This shows that during the spread of the coronavirus epidemic, the sudden increase in the frequency of searches for keywords related to sense of smell deserves the attention of epidemic surveillance agencies.

Other studies look at the forecast of epidemic trends. Ortiz-Martínez et al. (10) present the evaluation results of the relationship between Colombian COVID-19 cases and Google searches. They find that after the first case in the country, search volume begins to increase significantly. After this, there is a high correlation between the incidence of COVID-19 in Colombia and Google searches. Although Internet searches and social media data are related to traditional surveillance data, Internet search data can predict the outbreak of a disease several days or weeks in advance. Analysis shows that Google Trends can potentially determine the appropriate time and place to implement risk communication strategies for the affected population. Gozgor et al. (11), Ashraf (12), Ortiz-Martínez et al. (10), Fang et al. (13), Sharif et al. (14), Wang et al. (15), and Wu et al. (16) believe that in countries that lack diagnostic and surveillance capabilities, Google Trends or Baidu index can be used to monitor search changes related to COVID-19 and stock markets.

Sulyok et al. (17) point out that the use of Internet search data can improve the accuracy of COVID-19 pandemic disease modeling. It is believed that integrating Google Trends data into the distributed lag model can significantly improve the prediction quality of the disease model. But Springer et al. (6) also point out the limitations of the use of Google Trends, arguing that Google Trends can only represent the interest of the crowd and cannot clearly distinguish fear, worry, or pure interest. Therefore, researchers are cautioned to pay attention to this issue when using Google Trends.

Sousa-Pinto et al. (18) argue that the use of Google Trends has changed. In recent years, Google Trends has shifted from monitoring to predicting changes. Therefore, linking Google Trends with other data sources can help overcome the limitations of using only search information. The study by Springer et al. (6) also shows that the current population's main interest is in medical treatment. Apart from individual reports, people's interest in possible virus carriers or animal origins and repositories is also decreasing. For example, the authors find that the search term “COVID-19” and the search term “vaccine” have a high correlation, but the correlation with “pangolin” and “bat” is weak.

## Variable Description and Statistical Description

**Figure 1:**
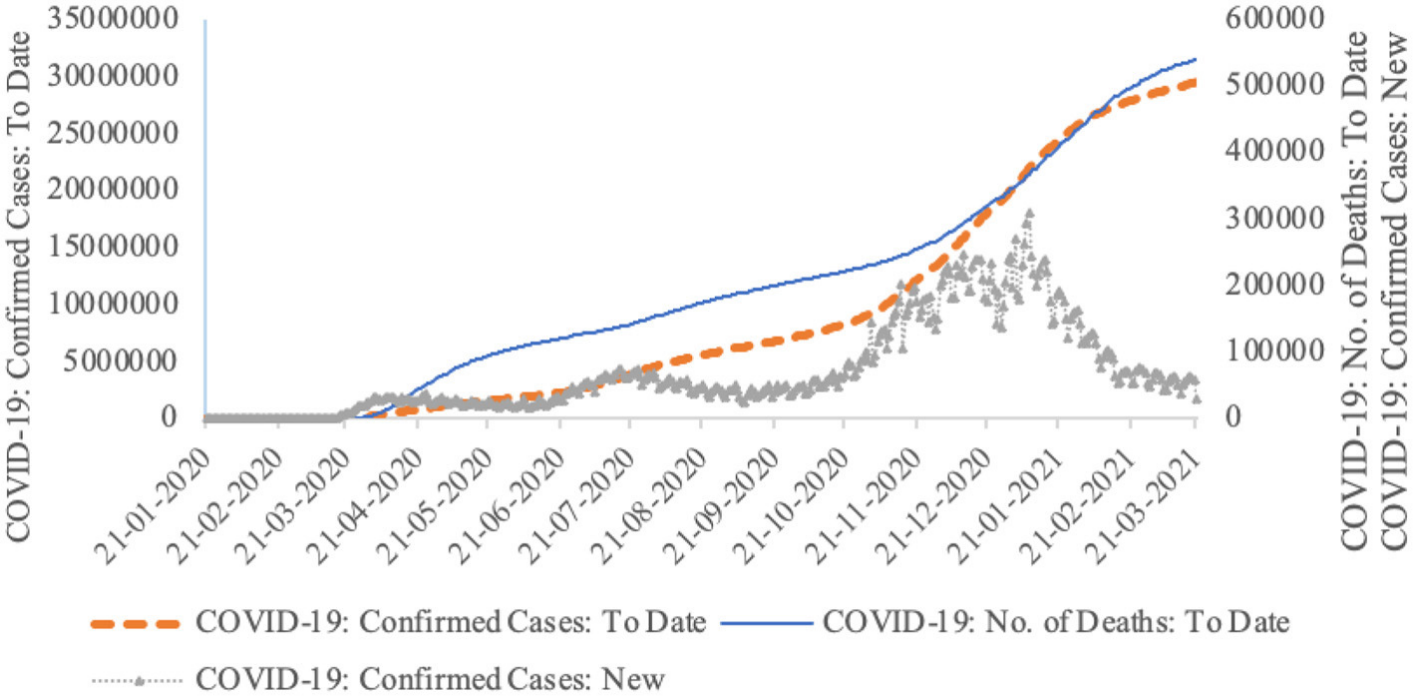
U.S. epidemic data released by Centers for Disease Control and Prevention.

As of March 23, 2021, the United States has become the country with the largest number of confirmed COVID-19 cases and deaths in the world, reaching 29.5 million and 543,000, respectively (Figure 1). Although the number of new cases in a single day has fallen sharply, it is still close to 30,000. Based on the availability and continuity of data, this article uses newly confirmed cases of COVID-19 as an alternative indicator of the U.S. epidemic, denoted by CONFIRMEDCASE. Data come from CEIC database.

Due to public concern about the possibility of becoming infected with COVID-19 during the epidemic, people try their best to engage in coronavirus surveillance or to search for relevant information. Therefore, we use the keyword search in Google Trends to express public sentiment. This article uses the keyword “COVID-19 test” to represent public sentiment concerning the epidemic. We use Baker et al. (4) to construct a newspaper-based infectious disease equity market volatility tracker to represent the volatility of infectious disease equities. We use INFECTIOUSEQUITY to express this. The statistical description of the main variables is shown in Table 1.

**Table 1:** Statistical description of the main variables.

	**CONFIRMEDCASE**	**COVID-19 TEST**	**INFECTIOUSEQUITY**
Mean	77498.46	51.24074	24.95998
Median	52847	47	21.283
Maximum	243448	100	65.931
Minimum	44	9	13.159
Std. Dev.	67254.04	20.57562	10.56136
Skewness	1.094862	0.661805	2.095552
Kurtosis	2.878847	2.930416	7.511875
Jarque-Bera	10.82152	3.952763	85.32532
Probability	0.004468	0.13857	0
Sum	4184917	2767	1347.839
Sum Sq. Dev.	2.40E+11	22437.87	5911.747
Observations	54	54	54

Google Trends data is on a weekly basis; therefore, our daily-based new confirmed cases and infectious disease equity market volatility tracker data must be averaged on a weekly basis. As such, part of the data contains a decimal point. Table 1 shows that the maximum number of newly confirmed cases is 243,448, which was obtained on January 3, 2021. The minimum value is 44, which was obtained on March 1, 2020, at the beginning of the epidemic. The maximum value of COVID-19 TEST data is 100, which was obtained on June 21, 2020. Although the epidemic in the United States was not very serious at the time, the southern states of the United States allowed companies to reopen, resulting in a surge in confirmed cases. Public concern and media propaganda caused searches to soar rapidly. The minimum value is 9, which is also obtained on March 1, 2020 at the beginning of the sample period. The maximum value of the infectious disease equity market volatility was obtained on March 15, 2020, and the minimum value was obtained on February 7, 2021.

## Empirical Research

The correlation between the main variables shows that confirmed cases are positively correlated with COVID-19 TEST. This shows that the more confirmed cases, the greater the public's attention to the epidemic and the more they are willing to retrieve COVID-19 TEST related information. There is a negative correlation between confirmed cases and the INFECTIOUSEQUITY variable, but the correlation between them is not strong.

Table 2 uses ADF and PP to investigate the unit root test results. The ADF and PP tests are based on the following assumptions: testing the null hypothesis of unit roots (non-stationary) and the alternative hypothesis of no unit roots (stationary). The model estimates the presence and absence of trends, levels, and first-order differences. When the level value contains a trend, the ADF and PP test results of the three variables reject the null hypothesis that the unit root is at the 1% significance level. This means that these series are not stationary at their level values. When the first-order difference does not include trend and intercept, all three variables pass the 1% significance test. This shows that the first difference of the three variables is a stationary time series.

**Table 2:** ADF and PP test results.

**Variables**	**ADF**		**PP**	
	**Level**	**1st difference**	**Level**	**1st difference**
	**Intercept**	**Without trend and intercept**	**Intercept**	**Without trend and intercept**
CONFIRMEDCASE	−2.451	−3.976^***^	−1.574	−3.825^***^
Covid-19 Test	−2.793^*^	−6.095^***^	−3.200^**^	−6.076^***^
INFECTIOUSEQUITY	−2.351	−8.243^***^	−2.268	−9.985^***^

***, **, and * *indicate the significance levels at 1%, 5%, and 10% levels, respectively*.

Table 3 shows the test results of Granger causality. We have selected an appropriate lag period according to the Akaike Information Criteria (AIC). The test results show that INFECTIOUSEQUITY does not Granger Cause CONFIRMEDCASE, rejecting the null hypothesis at the 1% level. Similarly, CONFIRMEDCASE does not Granger Cause INFECTIOUSEQUITY, rejecting the null hypothesis at the 1% significance level. This means that there is a two-way Granger causality between INFECTIOUSEQUITY and CONFIRMEDCASE. In other words, the increase in confirmed cases will cause fluctuations in epidemic equities. Similarly, the fluctuation of equity information about the epidemic also reflects the progress of confirmed cases. In sharp contrast, CONFIRMEDCASE does not Granger Cause COVID-19 TEST, rejecting the null hypothesis at a significance level of 10%, but the opposite is not true. COVID-19 TEST does not Granger Cause INFECTIOUSEQUITY, also rejecting the null hypothesis at a significance level of 10%, and vice versa.

**Table 3:** Granger causality test results.

**Null hypothesis**	**Obs**	**F-statistic**	**Prob**.
INFECTIOUSEQUITY does not Granger Cause CONFIRMEDCASE	52	10.1390	0.0002
CONFIRMEDCASE does not Granger Cause INFECTIOUSEQUITY		16.4139	4.00E-06
COVID-19 TEST does not Granger Cause CONFIRMEDCASE	52	1.08637	0.3458
CONFIRMEDCASE does not Granger Cause COVID-19 TEST		2.52758	0.0907
COVID-19 TEST does not Granger Cause INFECTIOUSEQUITY	52	2.78003	0.0723
INFECTIOUSEQUITY does not Granger Cause COVID-19 TEST		0.30440	0.739

The results of the cointegration test show that there is a cointegration relationship between the three variables. Therefore, we can conduct a VAR inspection and impulse response function analysis.

**Figure 2:**
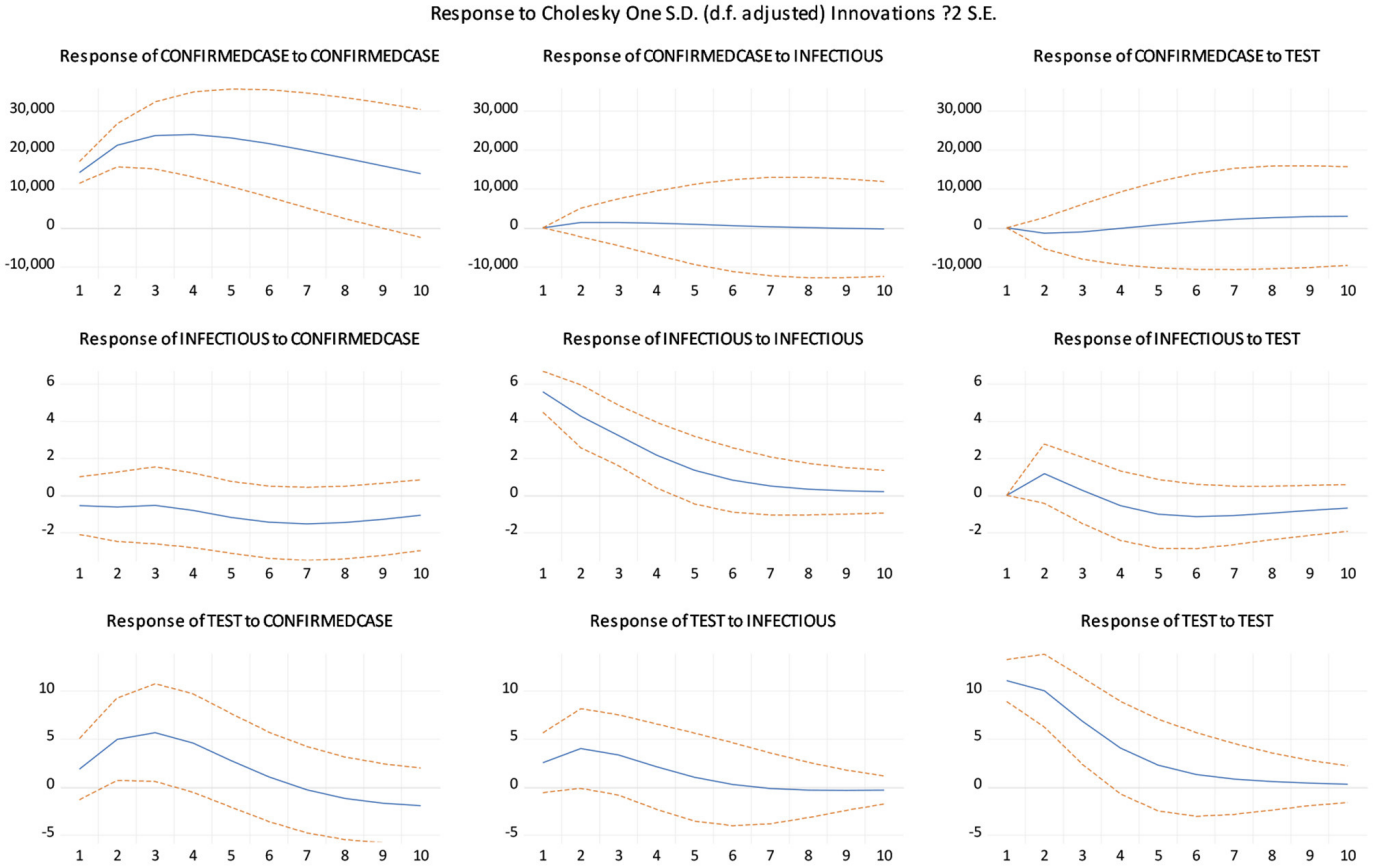
Impulse response function.

The impulse response function in Figure 2 mainly examines the impact of one variable in different lag periods on other variables. The calculation results show that the response of CONFIRMEDCASE to the INFECTIOUSEQUITY shock and the response of CONFIRMEDCASE to the COVID-19 TEST shock are relatively stable. However, the impact of CONFIRMEDCASE on itself first increases and then decreases, and there is a long lag period. INFECTIOUSEQUITY is always negative for the response from CONFIRMEDCASE. INFECTIOUSEQUITY's response to the shock from the COVID-19 TEST changed from positive to negative. The impact of INFECTIOUSEQUITY on itself continues to decline. The response of COVID-19 TEST to shocks from the other two variables is similar, rising first and then falling in both cases. However, the response of COVID-19 TEST from its own shock continues to decline.

## Conclusions and Policy Recommendations

This study empirically examines the relationship between the volatility of infectious disease equities, public sentiment, and the COVID-19 epidemic from the perspective of the United States. This study uses weekly data from January 3, 2020 to March 7, 2021. The research results show that the confirmed cases in the United States are positively correlated with COVID-19 TEST. This shows that the more confirmed cases, the greater the public's attention to the epidemic and the more willing they are to retrieve COVID-19 TEST related information. There is a negative correlation between confirmed cases in the United States and the INFECTIOUSEQUITY variable. In other words, as more cases are confirmed, the equities related to the epidemic will gain, thereby reducing the volatility of related equities; there is a negative correlation between the two. The results of the Granger causality test show that there is a two-way Granger causality relationship between INFECTIOUSEQUITY and CONFIRMEDCASE. In other words, the increase in confirmed cases will cause fluctuations in epidemic equities. Similarly, the fluctuation of equity information about the epidemic reflects the progress of confirmed cases. In sharp contrast, CONFIRMEDCASE can cause COVID-19 TEST. COVID-19 TEST will cause INFECTIOUSEQUITY, but the reverse is not true. That is, there is a one-way causal relationship between them. The impulse response function calculation results based on the VAR model show that the impulse response of the three variables from their own shock is stronger, but there is a longer lag period. The impact of the other two variables is relatively stable.

Based on the above research conclusions, we believe that real-time monitoring of epidemic trends will not only help determine the volatility of epidemic-related equities, but it will also help policy makers to intervene before major equity market volatility occurs, thereby preventing excessive equity market volatility. Secondly, monitoring the equity information of the epidemic can also help reveal undetected epidemics or the needs for medicines and anti-epidemic materials in specific areas or among groups of people, so as to provide targeted epidemic prevention and medical services for specific groups. In addition, providing effective COVID-19 test services in accordance with the epidemic's trends will also help control the epidemic in the United States and prevent the global spread of the epidemic (19). The number of testing services will also help determine the epidemic and the prosperity index of medical equities and industries in advance and improve the medical industry's ability to respond to the epidemic.

## Data Availability Statement

Publicly available datasets were analyzed in this study. This data can be found at: trends.google.com.

## Author Contributions

JM: writing-original draft. QS: writing—review and editing. JZ: resources. LW: investigation and software. RX: proofreading. CY: draft writing, design, and literature part. All authors contributed to the article and approved the submitted version.

## Conflict of Interest

The authors declare that the research was conducted in the absence of any commercial or financial relationships that could be construed as a potential conflict of interest.

## References

[1] Li Y Liang C Ma F Wang J. The role of the IDEMV in predicting European stock market volatility during the COVID-19 pandemic. Financ Res Lett. (2020) 36:101749. 10.1016/j.frl.2020.101749 32908465 PMC7467939

[2] Mazur M Dang M Vega M. COVID-19 and the march 2020 stock market crash. Evidence from SandP1500. Finan Res Lett. (2021) 38:101690. 10.1016/j.frl.2020.101690 32837377 PMC7343658

[3] Jiang B Liu Z Shen R Huang L Tong Y Xia Y. Have COVID-19-related economic shocks affected the health levels of individuals in the United States and the United Kingdom? Front Public Health. (2020) 8:611325. 10.3389/fpubh.2020.611325 33363099 PMC7755999

[4] Baker SR Bloom N Davis SJ Kost KJ Sammon MC Viratyosin T. The unprecedented stock market reaction to COVID-19. Rev Asset Pricing Stud. (2020) 10:742–758. 10.3386/w26945

[5] Kutlu Ö. Analysis of dermatologic conditions in Turkey and Italy by using Google Trends analysis in the era of the COVID-19 pandemic. Dermatol Ther. (2020) 33:e13949. 10.1111/dth.13949 32614116 PMC7361070

[6] Springer S Menzel LM Zieger M. Google trends reveals: focus of interest in the population is on treatment options rather than theories about COVID-19 animal origin. Brain Behav Immun. (2020) 87:134–5. 10.1016/j.bbi.2020.05.005 32387509 PMC7201235

[7] Strzelecki A. The second worldwide wave of interest in coronavirus since the COVID-19 outbreaks in South Korea, Italy and Iran: a Google Trends study. Brain Behav Immun. (2020) 88:950–1. 10.1016/j.bbi.2020.04.042 32311493 PMC7165085

[8] Heerfordt C Heerfordt IM. Has there been an increased interest in smoking cessation during the first months of the COVID-19 pandemic? A Google Trends study. Public Health. (2020) 183:6–7. 10.1016/j.puhe.2020.04.012 32388011 PMC7167577

[9] Walker A Hopkins C Surda P. Use of Google Trends to investigate loss-of-smell-related searches during the COVID-19 outbreak. Int Forum Allergy Rhinol. (2020) 10:839–847. 10.1002/alr.22580 32279437 PMC7262261

[10] Ortiz-Martínez Y Garcia-Robledo JE Vásquez-Castañeda DL Bonilla-Aldana DK Rodriguez-Morales AJ. Can Google trends predict COVID-19 incidence and help preparedness? The situation in Colombia. Travel Med Infect Dis. (2020) 37:101703. 10.1016/j.tmaid.2020.101703 32360323 PMC7187809

[11] Gozgor G Lau CKM Sheng X Yarovaya L. The role of uncertainty measures on the returns of gold. Econ Lett. (2019) 185:108680. 10.1016/j.econlet.2019.108680

[12] Ashraf BN. Stock markets' reaction to COVID-19: Cases or fatalities? Res Int Bus Financ. (2020) 54:101249. 10.1016/j.ribaf.2020.101249 PMC7244441 34170989

[13] Fang J Gozgor G Lau CKM Lu Z. The impact of Baidu index sentiment on the volatility of China's stock markets. Financ Res Lett. (2020) 32:101099. 10.1016/j.frl.2019.01.011

[14] Sharif A Aloui C Yarovaya L. COVID-19 pandemic, oil prices, stock market, geopolitical risk and policy uncertainty nexus in the US economy: fresh evidence from the wavelet-based approach. Int Rev Financ Anal. (2020) 70:101496. 10.1016/j.irfa.2020.101496 PMC7227524 38620230

[15] Wang J Lu X He F Ma F. Which popular predictor is more useful to forecast international stock markets during the coronavirus pandemic: VIX vs EPU? Int Rev Financ Anal. (2020) 72:101596. 10.1016/j.irfa.2020.101596 PMC7521353 38620312

[16] Wu W Su Q Li C Yan C Gozgor G. Urbanization, disasters, and tourism development: evidence from RCEP countries. Sustainability. (2020) 12:1221. 10.3390/su12031221

[17] Sulyok M Ferenci T Walker M. Google trends data and COVID-19 in Europe: correlations and model enhancement are European wide. Transbound Emerg Dis. (2020). 10.1111/tbed.13887. [Epub ahead of print]. 33085851

[18] Sousa-Pinto B Anto A Czarlewski W Anto JM Fonseca JA Bousquet J. Assessment of the Impact of media coverage on COVID-19-related google trends data: infodemiology study. J Med Internet Res. (2020) 22:e19611. 10.2196/19611 32530816 PMC7423386

[19] Gozgor G. Global evidence on the determinants of public trust in governments during the COVID-19. Appl Res Q Life. (2021). 1–20. 10.1007/s11482-020-09902-6. [Epub ahead of print]. 33564341 PMC7862976

